# BUILDING COMMUNITY-BASED PARTNERSHIPS TO PROMOTE DIVERSE, INTERGENERATIONAL SERVICE-LEARNING OPPORTUNITIES

**DOI:** 10.1093/geroni/igac059.037

**Published:** 2022-12-20

**Authors:** Jason Garbarino, Janelle Sarnevitz

**Affiliations:** University of Vermont, Burlington, Vermont, United States; University of Vermont, Burlington, Vermont, United States

## Abstract

The next generation of healthcare providers require experiential learning opportunities which incorporate participation from historically marginalized, diverse populations. A longstanding interprofessional gerontology service-learning program with a measured ability to positively influence student perspectives working with older adults while reducing rates of social isolation among older participants, underwent recent changes in the recruitment of older adults to ensure increased participation from historically marginalized populations. Building partnerships with community-based organizations serving BIPOC, LGBTQ+, and rural older adults played an integral role in building a more diverse participant group for shared, intergenerational learning. Opportunities for student reflection via group debriefs and individual, written reflections promote a greater understanding and preparedness to work with diverse older adult populations.

